# 

**Published:** 1911-09-15

**Authors:** 


					﻿THE
DENTAL
REGISTER
Edited by Dr NELVILLE S. HOFF
ANN ARBOR, MICH.
VOL.LXV SEPTEMBER, 1911 NO. 9.
THE SAMUEL A. CROCKER CO., Publishers
OHIO DENTAL AND SURGICAL DEPOT
35 W. FIFTH STREET, CINCINNATI, 0.
PRICE, ONE DOLLAR A YEAR
Entered at the Post-Office at Cincinnati, O , as second-class mall matter
				

